# Absence of Neu5Gc and Presence of Anti-Neu5Gc Antibodies in Humans—An Evolutionary Perspective

**DOI:** 10.3389/fimmu.2019.00789

**Published:** 2019-04-30

**Authors:** Meghan O. Altman, Pascal Gagneux

**Affiliations:** ^1^Department of Pathology, Biomedical Research and Training Facility 2, Glycobiology Research and Training Center, University of California, San Diego, La Jolla, CA, United States; ^2^Department of Anthropology, University of California, San Diego, La Jolla, CA, United States

**Keywords:** sialic acid, Neu5Gc, Neu5Ac, xenoglycan, anti-glycan antibodies, xeno-antigen

## Abstract

The glycocalyx of human cells differs from that of many other mammals by the lack of the sialic acid N-glycolylneuraminic acid (Neu5Gc) and increased abundance of its precursor N-acetylneuraminic acid (Neu5Ac). Most humans also have circulating antibodies specifically targeting the non-human sialic acid Neu5Gc. Recently, several additional mammalian species have been found to also lack Neu5Gc. In all cases, loss-of-function mutations in the gene encoding the sialic acid-modifying enzyme CMAH are responsible for the drastic change in these species. Unlike other glycan antigens, Neu5Gc apparently cannot be produced by microbes, raising the question about the origin of these antibodies in humans. Dietary exposure and presentation on bacteria coating themselves with Neu5Gc from the diet are distinct possibilities. However, the majority of the non-human species that lack Neu5Gc do not consume diets rich in Neu5Gc, making it unlikely that they will have been immunized against this sialic acid. A notable exception are mustelids (ferrets, martens and their relatives) known for preying on various small mammal species rich in Neu5Gc. No studies exist on levels of anti-Neu5Gc antibodies in non-human species. Evolutionary scenarios for the repeated, independent fixation of *CMAH* loss-of-function mutations at various time points in the past include strong selection by parasites, especially enveloped viruses, stochastic effects of genetic drift, and directional selection via female immunity to paternal Neu5Gc. Convergent evolution of losses of the vertebrate-specific self-glycan Neu5Gc are puzzling and may represent a prominent way in which glycans become agents of evolutionary change in their own right. Such change may include the reconfiguration of innate immune lectins that use self-sialic acids as recognition patterns.

## Introduction

**Figure 1:**
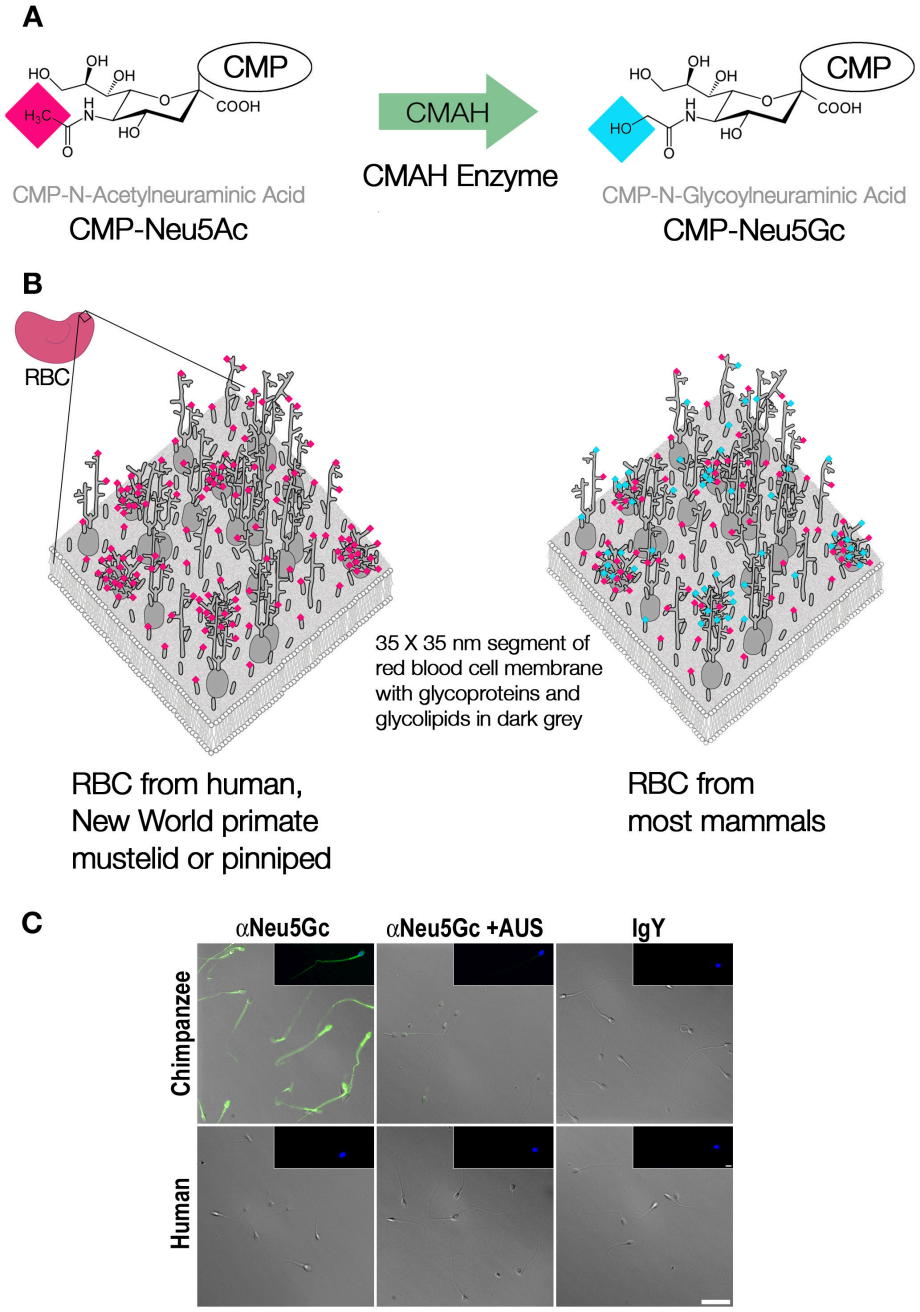
Modification of CMP-Neu5Ac to CMP-Neu5Gc. **(A)** The enzyme CMAH, encoded by a single gene in all mammals, catalyzes the derivatization of Neu5Ac to Neu5Gc in the form of their sugar nucleotides, cytidine monophosphate (CMP). **(B)** Due to the large number of sialic acids terminating many of the glycan chains on the glycocalyces of most cells, the loss of function of the *CMAH* gene leads to a drastic change in the molecular identity or “flavor” of the glycocalyx, as indicated by a small fraction of a red blood cell membrane, redrawn and modified from Viitala and Järnefelt (7). **(C)** Micrographs showing green immunofluorescent staining of Neu5Gc on chimpanzee but not human sperm cells stained with affinity purified chicken anti-Neu5Gc IgY antibody and fluorescent secondary, controls include sialidase treated or anti-IgY secondary antibody alone, nuclei stained by DAPI (blue) reprinted from Ghaderi et al. (8) with permission.

**Figure 2:**
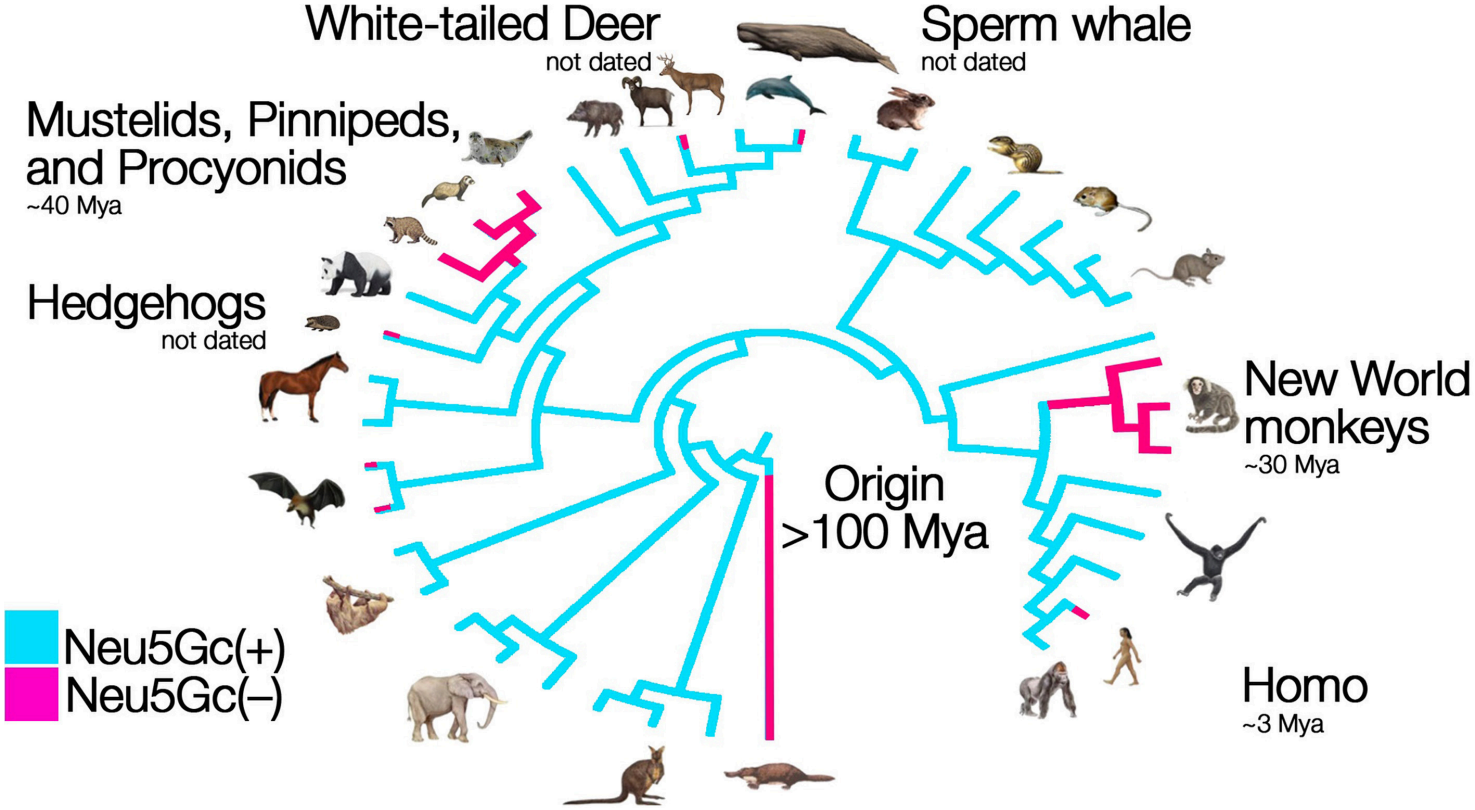
Parallel evolution and loss of an innate self-signal. Humans cannot synthesize Neu5Gc, because human *CMAH* was inactivated over two million years ago (red). The inactivating mutation apparently fixed rapidly after originating, which suggests that the loss could have been adaptive—driven by pathogen avoidance, reproductive conflict, or a combination of the two. Independent losses of Cmah function have recently been found in New World Primates, Mustelids and several other groups. Figure modified from Springer and Gagneux (9). In some lineages, such as bats and toothed whales, only certain species lost the capacity to make Neu5Gc (indicated by lines that are both blue and red).

The glycocalyx of all vertebrate cells is decorated with abundant terminal sialic acids. These acidic nine-carbon backbone sugars cap the ends of tens to hundreds of millions of glycan chains per cell. In mammalian species and other vertebrates, the sialic acids *N*-acetylneuraminic acid (Neu5Ac) and its derivative *N*-glycolylneuraminic acid (Neu5Gc) are the two most common forms, each a family of molecules with various modifications of the canonical, 9-carbon monosaccharide (1). Until recently, humans were the only mammalian species known to lack the sialic acid Neu5Gc, as our lineage fixed the loss-of-function mutation affecting the single copy *CMAH* gene that encodes the sialic acid-modifying enzyme CMAH over 2 million years ago in the lineage leading to *H. sapiens* (2, 3). More recently, several other species of mammals have been documented to also lack Neu5Gc due to ancient mutations fixed over 30 million years ago in these lineages (4–6). The loss of function of the CMAH enzyme prevents the modification of the precursor monosaccharide to the derived sialic acid Neu5Gc (in their respective sugar-nucleotide form, CMP-Neu5Ac and CMP-Neu5Gc). As illustrated in Figure 1, the lack of this enzymatic function can lead to drastic changes in the molecular composition of the glycocalyx of cells throughout the body. Recent evidence has shown that humans are not alone in this loss, instead several other species of mammals have independently fixed different loss-of-function mutations of their *Cmah* gene at various time depths during evolution, leading to loss of Neu5Gc in entire lineages or just individual species (6). These losses have occurred through exon deletion, premature stop codons, or frameshift mutations in the gene encoding the CMAH enzyme (4–6). The picture emerging is that of a phylogeny of mammals punctuated with taxa that have lost the capacity to synthesize Neu5Gc (Figure 2). These taxa include New World primates (>100 species of South and Central American primates known as *Platyrrhynes*), *Mustelidae* (57 species of small carnivores including ferrets, martens and weasels), pinnipeds (33 species comprising seals, sea-lions and walruses), *Procyonidae* (~15 species including racoons, ring-tails and coatis,) hedgehogs (17 species), bats from at least two different lineages, sperm whale (a single species), and white-tailed deer (a single species) (4–6). For most of these groups, only a few representative taxa and a few individuals have been studied at the genomic level, and so there is the possibility that the *Cmah* gene remained intact or polymorphic in some of the taxa.

An obvious prediction is that additional taxa with inactivated *Cmah* genes will be discovered as additional complete genome sequences are obtained. These cases of convergent molecular evolution result in an overall reconfiguration of the outermost layer of the glycocalyx, now lacking Neu5Gc and carrying an excess of Neu5Ac, given that human and non-human cells retain comparable levels of sialic acid (10) (see Figure 1B for red blood cells and Figure 1C for sperm cells). Among the many functions of the glycocalyx, molecular identity is paramount (11–13). The molecular patterns, as defined by composition and structure of the glycocalyx have evolved into self-associated molecular patterns (SAMPs) (14), that contribute to efficient surveillance by innate immune receptors including complement factor H and Siglecs, which can inhibit immune response upon engagement with SAMPs (14, 15). Losing Neu5Gc would dramatically alter self-recognition. This would have required evolving altered receptor specificities, affinities, and knock-on effects in signaling pathways due to altered engagement of innate receptors. The biochemical impact of the altered sialome on the human glycocalyx could have had many other effects, including changes in inflammation and metabolism (16, 17).

Another potential consequence are autoreactive antibodies produced against the lost sialic acid. Indeed, despite the absence of endogenous Neu5Gc, experimental studies in humans and in *Cmah*^(−/−)^ mice have revealed that dietary Neu5Gc, in both free and glycoconjugate-bound forms, can become incorporated into tissues in trace amounts. This incorporation occurs especially in tissues with rapid growth and/or turnover rates, including epithelia, endothelia, fetal tissues, and carcinomas (18–20). It has also been established that all humans have various levels of circulating antibodies specific for glycans carrying this foreign molecule, essentially making Neu5Gc a “xeno-autoantigen,” which can cause “xenosialitis,” an inflammation due to reaction against a xeno-sialic acid that is now part of “self” molecules (21–24). Surprisingly, even humans on diets extremely rich in Neu5Gc do not appear to accumulate beyond trace levels of this dietary xenoglycan.

How ingested Neu5Gc becomes incorporated into the human body remains incompletely understood. There is evidence that Neu5Gc is converted to GalNGc and can then be incorporated into the glycosaminoglycan chondroitin sulfate, an important component of extra-cellular matrices and skeletal bone (25). This incorporation has recently allowed the identification of GalNGc in bones and in fossilized bones as old as 3 million years, opening the possibility to study ancient glycomes of extinct hominins (26). There is much ongoing research to understand the potential effects of incorporation of dietary xeno-sialic acid and targeting antibodies against xeno-sialic acid, xenosialitis in the context of cancer and autoimmunity and even unexplained infertility, where chronic immune reactions to incorporated xenoglycans could contribute to xenosialitis (22–24, 27–29). Aside from humans, natural levels of anti-Neu5Gc antibodies in other species lacking Neu5Gc have not been studied to date. However, anti-Neu5Gc antibodies have been seen in chickens, where antibodies can be efficiently generated upon immunization (18) and are the basis of immune assays for the detection of Neu5Gc in human samples (30).

## Natural Immunization Against NEU5GC-glycans

There are four main differences between immunization against Neu5Gc and other xenoglycans, such as the disaccharide alpha-Gal, or alloglycans such as ABO oligosaccharide antigens. First, in the case of other xenoglycans, immunization against the missing, terminal “self”-glycan is thought to be caused by encounters with microbial glycans with the same structure (31, 32). Considering that the synthesis of endogenous Neu5Gc has never been documented for any microbe, it would appear unlikely that this microbial priming method occurs for Neu5Gc (33, 34). Despite the apparent lack of Neu5Gc synthetic capacity in microbes, however, at least one microbe, Non-typeable *Haemophilus influenzae* (NTHi), can scavenge dietary Neu5Gc and incorporate it into its own glycolipids. There is evidence that young human infants are “xeno-autoimmunized” against Neu5Gc by early *H. influenzae* infection and this method has also been utilized for experimental immunization of *Cmah*^(−/−)^ mice in the laboratory (35). Immunization thus seems to depend on diets rich in Neu5Gc from red meats or certain marine sources (fish eggs or echinoderms (27, 36). Secondly, unlike other xenoglycans, it is important to stress that the monosaccharide Neu5Gc itself is immunogenic, none of the constituent monosaccharides of alpha-Gal (galactose) or ABO antigens (fucose, galactose, N-Acetylgalactosamine, and N-Acetylglucosamine) are foreign to individuals lacking these structures and once ingested, they are incorporated as non-antigenic glycans or metabolized (37). The antigenicity of other xenoglycans, is largely determined by glycosidic linkages, rather than by the nature of the monosaccharide: galactose-alpha-1,3-galactose for alpha-Gal; fucose-alpha-1,2-galactose for the H antigen; H antigen with N-Acetylgalactosamine for A antigen; or H antigen with alpha-1,3-galactose for B antigen. Thirdly, unlike the other immunogenic glycans, Neu5Gc can be part of numerous different antigens depending on the identity of the sialoglycoconjugate they occur on. Finally, sialic acids are one to several orders of magnitude more abundant than either alpha-Gal or ABO glycans (38, 39). These three differences: dietary origin, antigenicity of the monomer itself, and ubiquity/abundance on the cell surface make Neu5Gc a unique antigen, whose loss may lead to wide-ranging physiological effects (37, 38).

While humans have many dietary sources for Neu5Gc, among the New World primates, there are very few species that eat vertebrate meat. Capuchin monkeys (genera *Cebus* and *Sapajou*) are known to prey on young coati (40), relatives of racoons belonging to the family of *Procyonidae*, and on lizards or birds, but these prey species all lack endogenous Neu5Gc (5, 41). It is thus very unlikely that these New World primates are immunized against Neu5Gc in the wild, but captive capuchin monkeys may be exposed to Neu5Gc through monkey chow containing red meat (Primate Info Net, University of Wisconsin). Hedgehogs and other insectivores, consume mostly insect prey that lack sialic acids and thus can be safely expected not to be naturally immunized against Neu5Gc (6). The same can be said for the different bat species that lack Neu5Gc, as these all feed on insects, fruit, or nectar (42). Pinnipeds (seals, sea-lions and walruses) are all strict carnivores and some of their prey include fish and marine invertebrates that could contain Neu5Gc (43). Studies of pinniped immune responses to sialic acids are urgently needed. The one species of whale also lacking Neu5Gc is the sperm whale (*Physeter catodon*) (6), whose diet consists mostly of giant squid and other cephalopods (squid and octopus) with occasional fish (44). Again, such a diet is unlikely to expose sperm whales to large amounts of Neu5Gc (45), leading to the prediction that they will not have circulating antibodies against the xenoglycan. Mustelids are the one group of species for which it can be assumed that dietary exposure and immunization occurs, as they are all known to feed on a variety of small mammals and vertebrates (46).

## Evolutionary Mechanisms for the Fixation of Loss-of-function Mutation

The loss-of-function mutations of the *Cmah* gene are by definition recessive, as one copy of the functional gene suffices to generate a Neu5Gc positive phenotype in a diploid organism.

### Balancing Selection Maintaining Polymorphisms

Some animals, including several dog and cat breeds, are polymorphic for Neu5Gc expression. While overall tissue expression is thought to be low, expression on blood cells in these animals can be high (47, 48). Polymorphisms involving Neu5Gc on the ganglioside GD3 exist in felids and are called AB blood groups in domestic cats (not related to primate ABO blood groups), where cats lacking Neu5Gc-GD3 have circulating antibodies specific for Neu5Gc (47). Dog breeds also differ in their expression of Neu5Gc on red blood cell glycolipids (48).

Due to the recessive nature of loss of function mutations, their increase in frequency within a population must be mediated by selection on homozygous carriers, who have fitness advantages conferring higher survival and/or reproductive success. For example, selection for polymorphisms involving a loss-of-function mutation could be based on the accompanying ablation of the glycan used as a receptor by pathogens (49–52).

Examples of Neu5Gc specific pathogens abound including the protozoan malaria parasite *P. reichenowi* (53), the swine pathogen *E. coli K99* (54) and the macaque monkey virus *SV40* (55).

**Figure 3:**
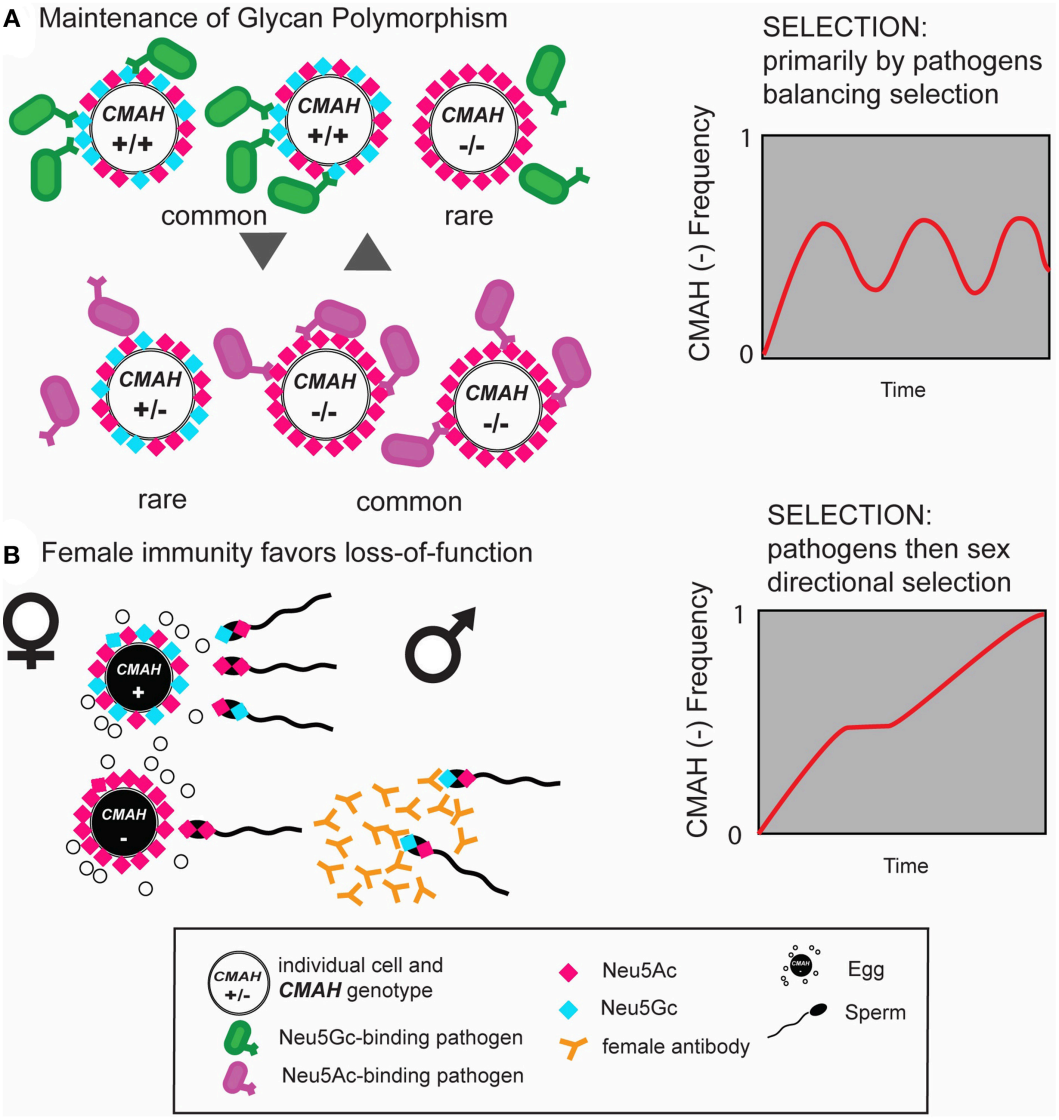
Schematic of the interplay of natural and sexual selection acting on cell-surface sialic acids. **(A)** Natural selection by pathogens recognizing and exploiting Neu5Gc (blue diamond) as a receptor on host target cells can select for mutant *CMAH*^(−)^ alleles that abolish expression of Neu5Gc in homozygote individuals and prevent infection. Such homozygous null individuals have only Neu5Ac on their cells (red diamonds) and at higher frequencies would be targeted by other pathogens adapted or adapting to the host glycan change (magenta). This equilibrium would result in maintenance of glycan polymorphism by balancing selection. **(B)** Anti-Neu5Gc antibody-expressing *CMAH*^(−/−)^ females, immunized by dietary consumption of Neu5Gc rich food (red meat) or by sperm antigens containing Neu5Gc, favor loss-of-function alleles on sperm due to reproductive incompatibility with *CMAH*^(−/+)^ or *CMAH*^(+/+)^ males expressing Neu5Gc on their sperm. Once the frequency of the *CMAH*^(−)^ allele reaches a certain level, this process can drive the fixation of the *CMAH*^(−)^ allele in a population via directional selection. Figure modified from Ghaderi et al. (8).

In contrast a number of human-specific pathogens evolved specificity for Neu5Ac, including the causative agent of human malignant malaria *P. falciparum*,the toxins of cholera agent *V. cholerae* (56) and typhoid fever agent *S. typhi* (57), and most influenza A viruses (58). Loss-of-function mutations, especially in polymorphic populations, could also provide partial protection from enveloped viruses that bear the antigenic glycan acquired from the cell membrane of the previous, Neu5Gc positive host. The latter mechanism would be analogous to such protection in alpha-Gal negative Old World primates (59–61) and across ABO mismatched humans (62–65). Such protective mechanisms are thus observed both, between species and within species with existing (balanced) polymorphisms. Balanced polymorphisms are maintained by frequency-dependent selection, i.e., selection favoring the rare variants, thus preventing their extinction but also preventing their fixation (see Figure 3) (66). Such dynamic co-evolutionary processes between pathogens and their hosts are the inspiration behind the terms evolutionary arms race and Red-Queen effects (15, 67).

Fixation of the loss-of-function allele, on the other hand, could happen either via directional selection or genetic drift, where small founder populations consist mostly of homozygous carriers of the loss-of-function mutation. First defined using plants in 1962 (68) and recently applied to primates by Galili (65), the idea of “catastrophic selection” combines these ideas with very strong selection. It is not clear how such “catastrophic selection” differs from short episodes of strong selection, possibly accompanied by demographic bottlenecks, which could also result in the fixation of loss-of-function mutations. Alternatively, selective pressure for *Cmah* loss-of-function could occur through reproductive conflict as discussed below.

### Female Immune-Mediated Selection Against Paternal Neu5Gc

Mammalian sperm are highly sialylated as a mechanism to enhance sperm survival and function along the perilous journey through the female reproductive tract to the site of fertilization in the oviduct (69–71). Mammals make anti-sperm antibodies when directly exposed to sperm (72). Major human sperm antigens include, highly sialylated GPI-anchored glycoproteins such as CD52 (73, 74), which in males that have a functional *CMAH* allele, carry Neu5Gc (75). Theoretically, immunization of females homozygous for the loss-of-function allele of *Cmah* could occur via insemination by males that have Neu5Gc-bearing sperm. Indeed, we have shown experimentally, using *Cmah*^(−/−)^ mice immunized against Neu5Gc, that their immune response against Neu5Gc bearing sperm severely reduces female fertility (8). In a further study, we demonstrated that Neu5Gc bearing sperm, both, sperm from either wild type mice or from *Cmah*^(−/−)^ *mice exposed to seminal fluid from wildtype mice containing Neu5Gc-rich CD52*, are both targeted by antibodies and are increasingly phagocytosed by female uterine immune cells (75). These insights have potential relevance for human fertility where Neu5Gc or anti-Neu5Gc antibodies in the reproductive tract are common among infertility patients, but not healthy controls (29).

In addition to blocking fertilization, it is possible that anti-Neu5Gc immunity from a primed *CMAH*^(−/−)^mother (29) could also negatively affect a *CMAH*^(+/−)^embryo or fetus in a manner similar to hemolytic diseases of the newborn caused by ABO glycan mismatches.

**Figure 4:**
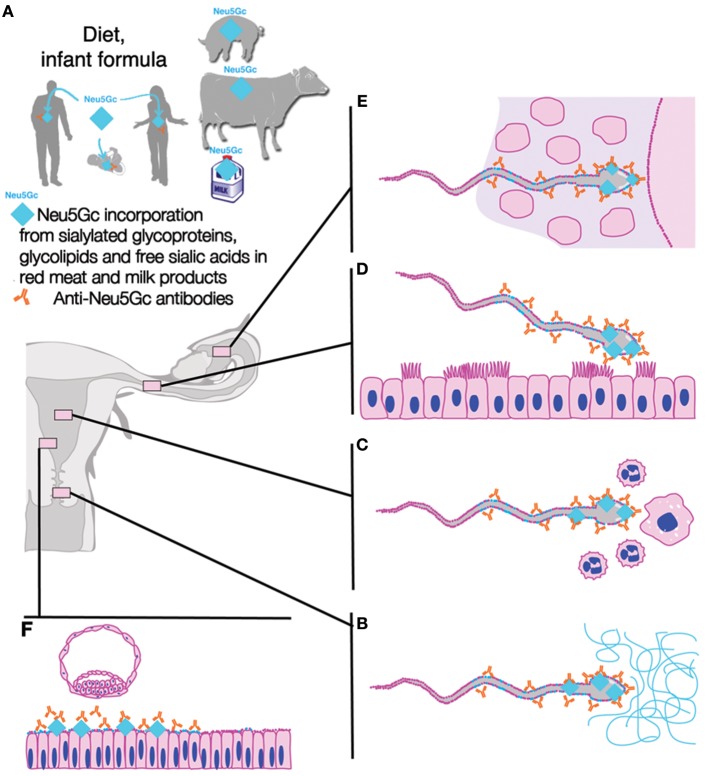
Exogenous (dietary) Neu5Gc and anti-Neu5Gc antibody as contributing factors to unexplained human infertility. The combination of incorporated dietary xenoglycan Neu5Gc (even in trace amounts) from red meat and milk products including cow milk-based infant formula and anti-Neu5Gc antibodies in males or females **(A)** could have deleterious consequences on human fertility via a number of potentially additive mechanisms which include: **(B)** coating of sperm by male and/or female anti-Neu5Gc antibodies, interference with sperm function, including passage through cervical mucins and/or other restrictive parts of the female reproductive tract such as the utero-tubal junction, **(C)** increased sperm death via female cellular and humoral immunity, where most sperm are killed by female immunity, **(D)** interference with sperm capacitation when sperm membranes get dynamically reconfigured, **(E)** interference with sperm penetration of vestment, interference with sperm-egg interactions, **(F)** interference with endometrial decidualization and receptivity resulting in reduced success of implantation. Figure modified from Ma et al. (75) with permission.

Reproductive xenosialitis could thus be a plausible mechanism mediating directional selection, leading to the fixation of the loss-of-function allele in the population, irrespective of the mechanism(s) involved for the initial selection favoring the mutation (see Figure 4).

## Conclusions and Perspectives

It is interesting that watershed events, such as the loss of Neu5Gc from the glycocalyx of human cells have occurred numerous times in many mammalian and other vertebrate species. These cases of convergent evolution represent precious opportunities for increased understanding of evolutionary processes. In some respects, Neu5Gc is an ideal self-molecule as it is “private” to vertebrates and, based on current data, has yet to be successfully mimicked by microbes. Against the background of this benefit, the loss of Neu5Gc appears paradoxical and may implicate strong selective regimes, either catastrophically caused by pathogens, or under directional sexual selection via female immunity to paternal xenoglycans. Massive genetic drift, or combinations of milder selection and founder events, can also not be excluded.

More information on species expected to encounter Neu5Gc in their diets, i.e., mustelids, pinnipeds, and humans, is needed to begin answering several outstanding questions in the field: For instance, what are the potential protective functions of anti-Neu5Gc antibodies in species that lack this sialic acid, especially as regards ongoing protection from cross-species infections by enveloped viruses bearing Neu5Gc on their viral envelopes? Or on the flip-side, what are the potential liabilities of anti-Neu5Gc antibodies due to autoimmunity against incorporated dietary Neu5Gc? Evolutionary events such as the ones discussed here exemplify how glycans, rather than representing the end result of different evolutionary histories and contingencies, can become an evolutionary force of their own and constrain future evolution of entire lineages including subsequent compensatory evolution of glycan binding immune receptors (15, 76).

## Author Contributions

MA and PG did the research and wrote the paper. PG produced the figures.

### Conflict of Interest Statement

The authors declare that the research was conducted in the absence of any commercial or financial relationships that could be construed as a potential conflict of interest.
